# Testosterone enhances mitochondrial complex V function in the substantia nigra of aged male rats

**DOI:** 10.18632/aging.103265

**Published:** 2020-05-23

**Authors:** Tianyun Zhang, Yu Wang, Yunxiao Kang, Li Wang, Hui Zhao, Xiaoming Ji, Yuanxiang Huang, Wensheng Yan, Rui Cui, Guoliang Zhang, Geming Shi

**Affiliations:** 1 Department of Neurobiology, Hebei Medical University, Shijiazhuang 050017, China; 2 Department of Sports Medicine, Hebei Sport University, Shijiazhuang 050017, China; 3 Department of Anatomy, Hebei Medical University, Shijiazhuang 050017, China; 4 Neuroscience Research Center, Hebei Medical University, Shijiazhuang 050017, China; 5 Hebei Key Laboratory of Forensic Medicine, Department of Forensic Medicine, Hebei Medical University, Shijiazhuang 050017, China

**Keywords:** testosterone, mitochondrial complex V, substantia nigra, aged male rats

## Abstract

Deficits in coordinated motor behavior and mitochondrial complex V activity have been observed in aged males. Testosterone supplementation can improve coordinated motor behavior in aged males. We investigated the effects of testosterone supplementation on mitochondrial complex V function in the substantia nigra (a brain region that regulates motor activity) in aged male rats. These rats exhibited diminished ATP levels, attenuated mitochondrial complex V activity, and reduced expression of 3 of the 17 mitochondrial complex V subunits (ATP6, ATP8 and ATP5C1) in the substantia nigra. Testosterone supplementation increased ATP levels, mitochondrial complex V activity, and ATP6, ATP8 and ATP5C1 expression in the substantia nigra of the rats. Conversely, orchiectomy reduced mitochondrial complex V activity, downregulated ATP6 and ATP8 expression, and upregulated ATP5C1, ATP5I and ATP5L expression in the substantia nigra. Testosterone replacement reversed those effects. Thus, testosterone enhanced mitochondrial complex V function in the substantia nigra of aged male rats by upregulating ATP6 and ATP8. As potential testosterone targets, these two subunits may to some degree maintain nigrostriatal dopaminergic function in aged males.

## INTRODUCTION

Endogenous testosterone and structurally related synthetic compounds notably impact the behavior of various organisms [1, 2]. Testosterone replacement effectively reverses the motor behavioral deficits of adult male rats with testosterone deficiency [3], and somewhat alleviates the motor and non-motor symptoms of men with Parkinson’s disease [4]. In the normal aging process, the functions of many tissues and organs progressively decline [5–7]. Testosterone levels gradually but eventually significantly decrease in aged men and aged male animals [8, 9].

The substantia nigra (SN), a brain region that controls motor behavior and is damaged by Parkinson’s disease, exhibits degeneration upon aging as the levels of dopamine, tyrosine hydroxylase and dopamine transporter in the nigrostriatal dopaminergic system decrease [10–12]. Testosterone supplementation can ameliorate the defects in the nigrostriatal dopaminergic system in aged male rats, possibly by enhancing mitochondrial function [13, 14]. In support of this notion, orchiectomy was found to reduce mitochondrial respiratory chain activity in the SN in adult male rats [14].

The brain is a highly differentiated organ with high energy requirements. It is primarily powered by adenosine triphosphate (ATP) produced by mitochondrial oxidative phosphorylation [15]. Mitochondrial dysfunction, characterized by excessive reactive oxygen species levels, reduced ATP levels and diminished mitochondrial respiratory chain activity, is involved in aging and age-related neurodegenerative diseases [16–19]. During the aging process, maintaining the normal function of the mitochondrial respiratory chain can enhance the survival of senescent neurons [20].

There are five complexes in the mitochondrial respiratory chain. Mitochondrial complexes I, II, III and IV transfer electrons to complex V to synthesize ATP [21]. In adult male rats, testosterone deficiency impairs mitochondrial function in the heart [22, 23] and downregulates the gene expression of mitochondrial complexes I, III and IV in the brain. Testosterone supplementation was found to restore the gene expression of complexes I, III and IV in the brains of castrated adult male rats [13, 14]. However, it is not known whether the subunits of mitochondrial complex V, the ATP generator, are also influenced by testosterone levels.

Mitochondrial complex V, also known as F_1_F_O_ ATP synthase, catalyzes the synthesis of ATP using energy from an electrochemical proton gradient derived from electron transport [24]. Mammalian mitochondrial complex V has 17 subunits, including two mitochondrial DNA (mtDNA)-encoded subunits (ATP6 and ATP8) and 15 nuclear DNA (nDNA)-encoded subunits [25, 26]. Complex V activity and subunit levels in certain tissues are reduced in aged male animals [27, 28]. In the present study, we investigated the effects of testosterone propionate (TP) supplementation on mitochondrial complex V activity and subunit levels in the SN of aged male rats and gonadectomized adult male rats, in order to detect potential testosterone targets that maintain nigrostriatal dopaminergic function in aged males.

## RESULTS

### TP supplementation ameliorated coordinated motor behavioral deficits in aged male rats

**Figure 1:**
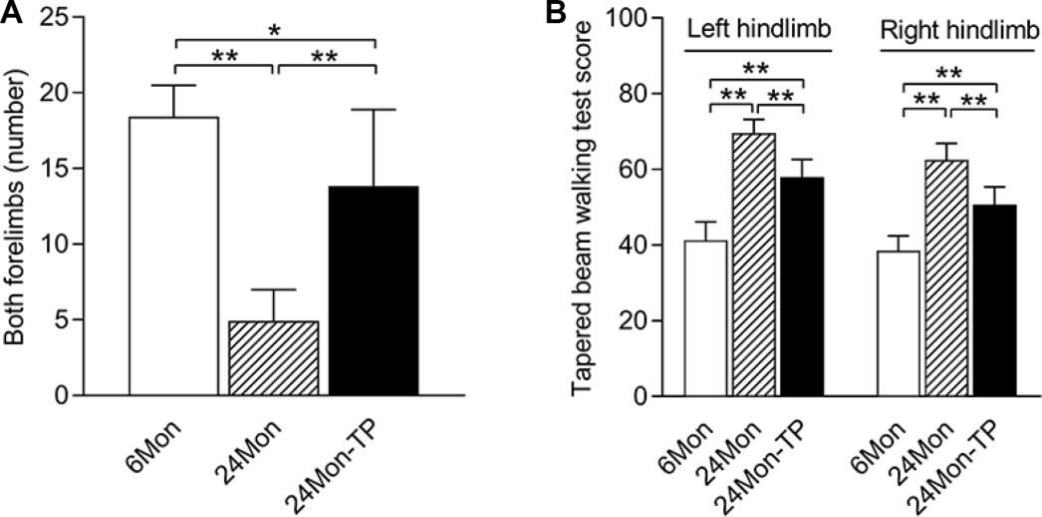
**TP supplementation ameliorated the coordinated motor behavioral deficits of aged male rats.** (**A**) Effects of TP supplementation on the number of times the aged male rats contacted the wall with both forelimbs during rearing. (**B**) Effects of TP supplementation on the tapered beam walking test scores of the hindlimbs of aged male rats. Data are expressed as the mean ± S.D. (n=12 rats/group). ^*^P<0.05, ^**^P<0.01.

We first performed cylinder tests and tapered beam walking tests to examine coordinated motor behavior in younger rats (6 months old, ‘6Mon’) and in aged rats (24 months old, ‘24Mon’) with and without TP supplementation. In the cylinder test, the number of times the rats contacted the wall with both forelimbs differed among the 6Mon, 24Mon and 24Mon-TP groups (Figure 1A, *P*<0.01). Post hoc analysis revealed that the number of times the rats touched the wall with both forelimbs was lower in the 24Mon group than in the 6Mon group (*P*<0.01), and greater in the 24Mon-TP group than in the 24Mon group (*P*<0.01). However, the number of times the rats touched the wall with both forelimbs in the 24Mon-TP group did not reach the level of the 6Mon group (*P<*0.05).

The tapered beam walking test scores also differed significantly among the 6Mon, 24Mon and 24Mon-TP groups (Figure 1B, left hindlimb, right hindlimb: *P<*0.01). The test scores for both the left and right hindlimbs were greater in 24Mon rats than in 6Mon rats (*P<*0.01), and were lower in 24Mon-TP rats than in 24Mon rats (*P<*0.01). However, the test scores for both the left and right hindlimbs in the 24Mon-TP group did not reach the level of the 6Mon group (*P<*0.01).

### TP supplementation increased ATP levels in the SN of aged male rats

**Figure 2:**
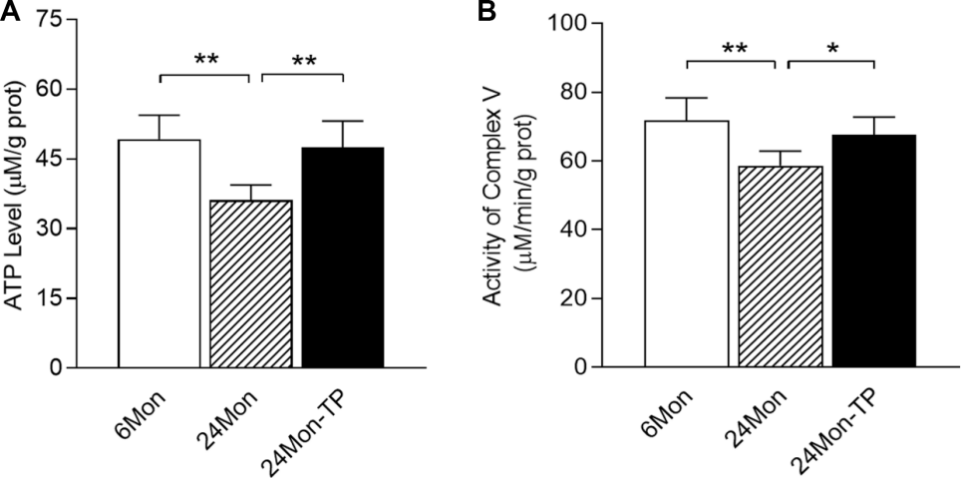
**Effects of TP supplementation on ATP levels and mitochondrial complex V activity in the substantia nigra of aged male rats.** (**A**) ATP levels. (**B**) Mitochondrial complex V activity. Data are expressed as the mean ± S.D. (n=6 rats/group). ^*^P<0.05, ^**^P<0.01.

We next measured ATP levels in the SN, and detected marked differences among the 6Mon, 24Mon and 24Mon-TP groups (Figure 2A, *P<*0.01). ATP levels in the SN were lower in 24Mon rats than in 6Mon rats (*P<*0.01). TP supplementation increased ATP levels in the SN of aged male rats (*P<*0.01) to the level of 6Mon rats.

### TP supplementation enhanced mitochondrial complex V activity in the SN of aged male rats

Considering the altered ATP levels in TP-treated aged male rats, we next assessed the effects of TP supplementation on mitochondrial complex V activity in the SN. Mitochondrial complex V activity in the SN differed significantly among the 6Mon, 24Mon and 24Mon-TP groups (Figure 2B, *P<*0.01). Mitochondrial complex V activity in the SN was lower in 24Mon rats than in 6Mon rats (*P<*0.01). TP supplementation of 24Mon rats enhanced mitochondrial complex V activity in the SN (*P<*0.05); in fact, there was no significant difference in mitochondrial complex V activity in the SN between 24Mon-TP rats and 6Mon rats.

### Single-nucleotide polymorphism (SNP) screening of mtDNA-encoded subunits of mitochondrial complex V in the SN

To determine whether mitochondrial complex V activity was altered due to DNA mutations, we screened the mtDNA-encoded subunits of mitochondrial complex V for SNPs, since mtDNA is more vulnerable to oxidative damage than nDNA. The DNA sequences of *ATP6* (Supplementary Datum 1) and *ATP8* (Supplementary Datum 2) in the SN displayed 100% identity among 6Mon, 24Mon and 24Mon-TP rats.

### Effects of TP supplementation on mitochondrial complex V subunit expression in the SN of aged male rats

**Figure 3:**
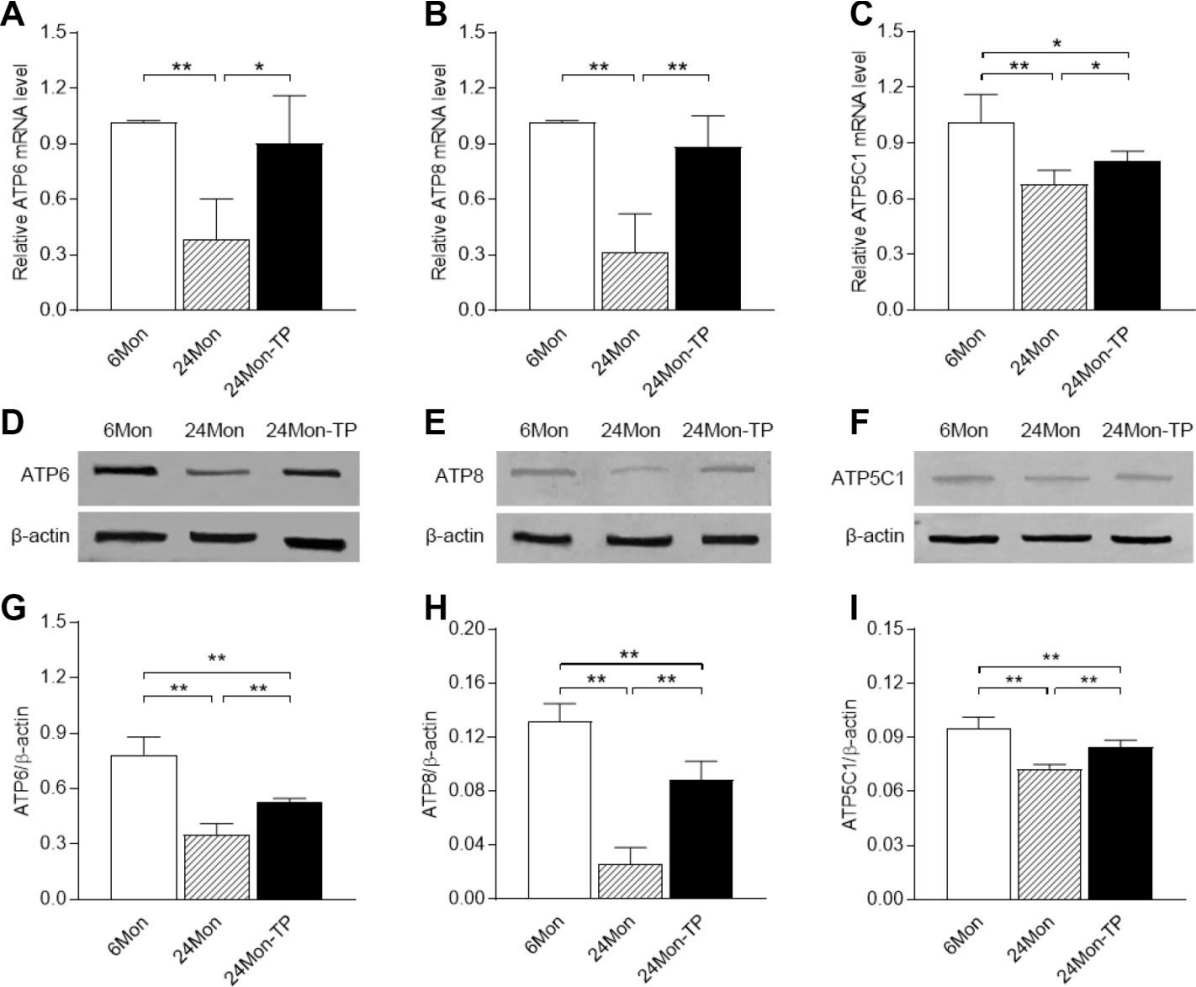
**Effects of TP supplementation on complex V subunit expression in the substantia nigra of aged male rats.** (**A**–**C**) The mRNA levels of *ATP6*, *ATP8* and *ATP5C1* were calculated using the 2^-ΔΔCt^ method. *GAPDH* was used as an internal control. (**D**–**F**) Representative Western blots of ATP6, ATP8 and ATP5C1 protein levels. (**G**–**I**) ATP6, ATP8 and ATP5C1 protein levels were quantified by comparing the band density of each protein to that of β-actin (endogenous control). Data are expressed as the mean ± S.D. (n=6 rats/group). ^*^P<0.05, ^**^P<0.01.

Based on the altered activity of mitochondrial complex V and the results of the SNP assay, we next analyzed the expression of mitochondrial complex V subunits. ATP6, ATP8 and ATP5C1 mRNA and protein levels in the SN were lower in 24Mon rats than in 6Mon rats (Figure 3, *P<*0.01), and were greater in 24Mon-TP rats than in 24Mon rats (mRNA: Figure 3A, *ATP6*, *P<*0.05; Figure 3B, *ATP8*, *P<*0.01; Figure 3C, *ATP5C1*, *P<*0.05. Protein: Figure 3G–3I, *P<*0.01). However, *ATP5C1* mRNA levels (Figure 3C, *P<*0.05) and ATP6, ATP8 and ATP5C1 protein levels (Figure 3G–3I, *P<*0.01) in the SN were still lower in 24Mon-TP rats than in 6Mon rats. There were no differences in the mRNA levels of the other subunits of mitochondrial complex V in the SN among 6Mon, 24Mon and 24Mon-TP rats (Table 1).

**Table 1:** Effects of TP supplementation on complex V subunit mRNA levels in the substantia nigra of aged male rats.

**Subunits**	**6Mon**	**24Mon**	**24Mon-TP**
ATP6	1.02±0.01	0.39±0.21^**^	0.90±0.26^#^
ATP8	1.02±0.01	0.31±0.21**	0.88±0.17^##^
ATP5A1	1.01±0.16	0.92±0.12	0.89±0.17
ATP5B	1.00±0.06	1.00±0.07	1.04±0.12
ATP5C1	1.01±0.15	0.68±0.08^**^	0.80±0.05^*#^
ATP5D	1.00±0.09	1.01±0.09	1.03±0.13
ATP5E	1.00±0.10	0.96±0.04	1.03±0.12
ATP5F1	1.01±0.17	1.03±0.18	1.04±0.12
ATP5G1	1.00±0.11	0.93±0.14	1.00±0.15
ATP5G2	1.01±0.11	1.05±0.08	1.04±0.08
ATP5G3	1.00±0.07	1.02±0.08	1.07±0.09
ATP5O	1.00±0.09	1.01±0.09	1.03±0.13
ATP5H	1.01±0.11	0.96±0.12	1.02±0.06
ATP5J	1.00±0.09	0.98±0.08	1.05±0.07
ATP5I	1.00±0.08	0.94±0.11	1.08±0.03
ATP5J2	1.00±0.11	1.03±0.09	1.05±0.10
ATP5L	1.01±0.13	1.11±0.12	0.99±0.16

Data were shown as mean ± S.D. (n=6 rats/group). ^*^P<0.05 versus 6Mon; ^**^P<0.01 versus 6Mon; ^#^*P*<0.05 versus 24Mon; ^##^*P*<0.01 versus 24Mon.

### Serum testosterone levels and body weights of TP-supplemented aged male rats

**Figure 4:**
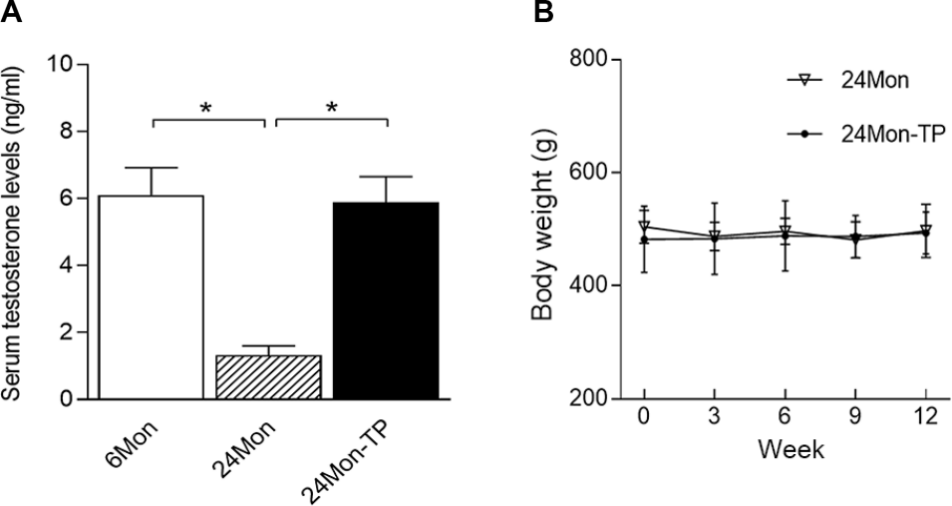
**Serum testosterone levels and body weights.** (**A**) Serum testosterone levels were significantly lower in 24Mon rats than in 6Mon rats. Supplementation of aged male rats with TP increased their serum testosterone concentrations to the level of 6Mon rats. (**B**) No differences in body weight were detected between 24Mon-TP and 24Mon rats. Data are expressed as the mean ± S.D. (n=12 rats/group). ^*^P<0.01.

Serum testosterone levels differed significantly among the 6Mon, 24Mon and 24Mon-TP rats (Figure 4A, *P*<0.01). Serum testosterone levels were significantly lower in 24Mon rats than in 6Mon rats (*P*<0.01). Supplementation of aged male rats with TP increased their serum testosterone levels to those of 6Mon rats. No difference in body weight was found between 24Mon-TP rats and 24Mon rats (Figure 4B).

### Gonadectomy of adult male rats impaired mitochondrial complex V in the SN

**Figure 5:**
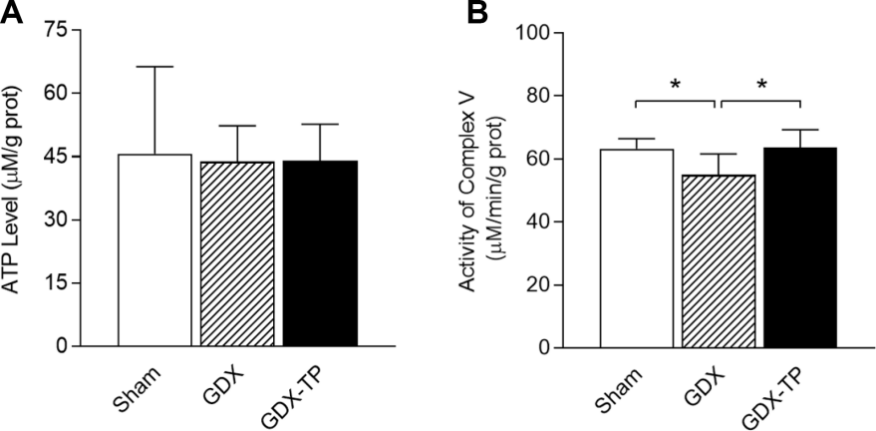
**Effects of gonadectomy and TP replacement on ATP levels and mitochondrial complex V activity in the substantia nigra of adult male rats.** (**A**) ATP levels. (**B**) Mitochondrial complex V activity. Data are expressed as the mean ± S.D. (n=6 rats/group). ^*^P<0.05.

**Figure 6:**
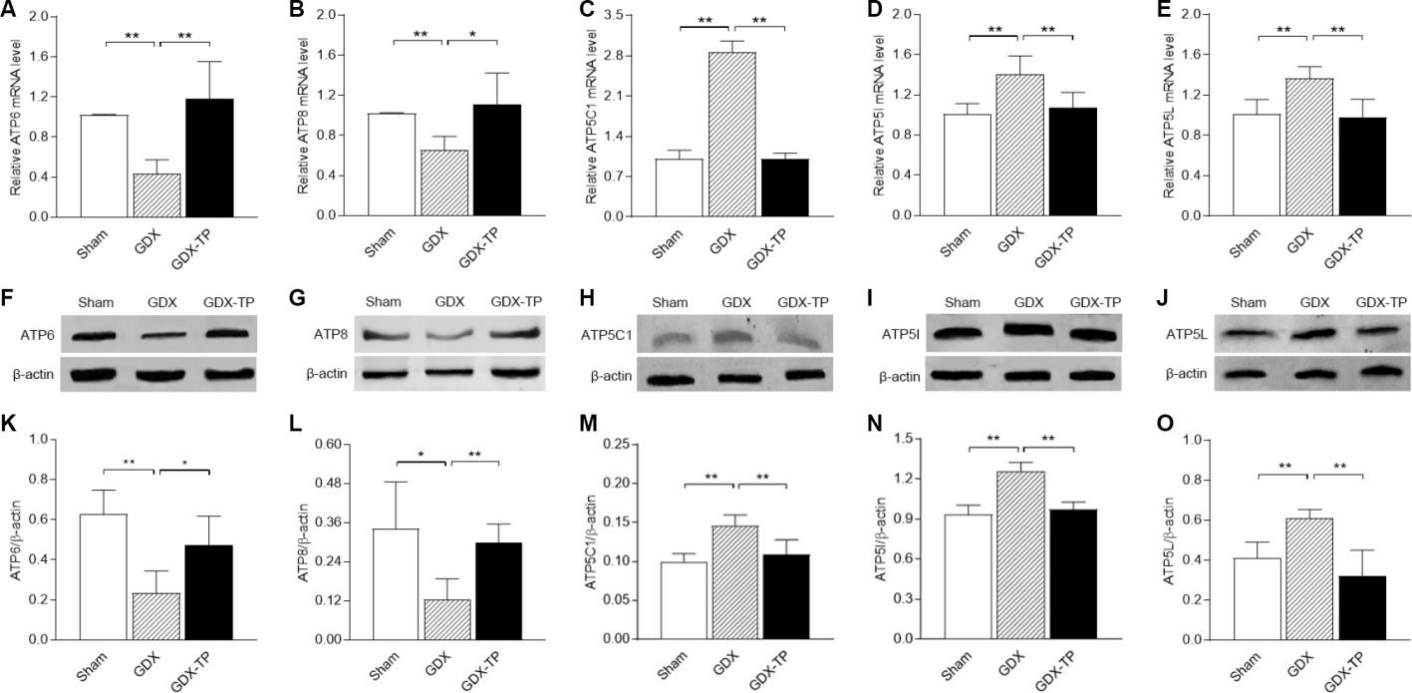
**Effects of gonadectomy and TP replacement on mitochondrial complex V subunit expression in the substantia nigra of adult male rats.** (**A**–**E**) The mRNA levels of *ATP6*, *ATP8*, *ATP5C1*, *ATP5I* and *ATP5L* were calculated using the 2^-ΔΔCt^ method. *GAPDH* was used as an internal control. (**F**–**J**) Representative Western blots of ATP6, ATP8, ATP5C1, ATP5I and ATP5L protein levels. (**K**–**O**) ATP6, ATP8, ATP5C1, ATP5I and ATP5L protein levels were quantified by comparing the band density of each protein to that of β-actin (endogenous control). Data are expressed as the mean ± S.D. (n=6 rats/group). ^*^P<0.05, ^**^P<0.01.

To rule out the influence of aging-related factors, we gonadectomized adult male rats and measured the ATP levels, mitochondrial complex V activity levels and mitochondrial complex V subunit mRNA and protein levels in the SN. Gonadectomy did not alter ATP levels in the SN of adult male rats (Figure 5A). However, gonadectomy reduced mitochondrial complex V activity (Figure 5B, *P<*0.05), downregulated ATP6 and ATP8 (mRNA: Figure 6A and 6B, *P<*0.01. Protein: Figure 6K and 6L, ATP6, *P<*0.01; ATP8, *P<*0.05) and upregulated ATP5C1, ATP5I and ATP5L (mRNA: Figure 6C–6E, *P<*0.01. Protein: Figure 6M–6O, *P<*0.01) in the SN of adult male rats. TP replacement reversed these effects. The mRNA levels of the other subunits of mitochondrial complex V in the SN did not differ among the sham-operated, gonadectomized and gonadectomized-TP rats (Table 2).

**Table 2:** Effects of gonadectomy and TP replacement on complex V subunit mRNA levels in the substantia nigra of adult male rats.

**Subunits**	**Sham**	**GDX**	**GDX-TP**
ATP6	1.02±0.01	0.44±0.14^*^	1.18±0.37^##^
ATP8	1.02±0.01	0.66±0.13^*^	1.11±0.31^#^
ATP5A1	1.01±0.14	1.04±0.09	1.02±0.11
ATP5B	1.01±0.14	0.89±0.07	0.96±0.10
ATP5C1	1.01±0.15	2.86±0.19^*^	1.01±0.10^##^
ATP5D	1.00±0.08	1.00±0.15	0.97±0.09
ATP5E	1.01±0.13	1.09±0.12	1.12±0.14
ATP5F1	1.00±0.11	0.98±0.08	0.99±0.11
ATP5G1	1.00±0.07	0.95±0.15	1.00±0.13
ATP5G2	1.01±0.13	1.03±0.12	1.00±0.10
ATP5G3	1.01±0.14	1.06±0.10	0.97±0.10
ATP5O	1.01±0.12	0.98±0.10	1.07±0.09
ATP5H	1.01±0.12	1.01±0.14	0.95±0.07
ATP5J	1.01±0.13	0.95±0.10	1.00±0.13
ATP5I	1.01±0.11	1.41±0.18^*^	1.07±0.15^##^
ATP5J2	1.01±0.11	1.01±0.09	1.02±0.15
ATP5L	1.01±0.15	1.37±0.12^*^	0.98±0.18^##^

Data were shown as mean ± S.D. (n=6 rats/group). ^*^P<0.01 versus sham; ^#^*P*<0.05 versus GDX; ^##^*P*<0.01 versus GDX.

## DISCUSSION

The present study demonstrated that testosterone supplementation of aged male rats ameliorated the deficits of mitochondrial complex V in the SN. In aged male rats, ATP levels were reduced, mitochondrial complex V activity was attenuated and the mRNA and protein levels of 3 of the 17 mitochondrial complex V subunits (ATP6, ATP8 and ATP5C1) were diminished in the SN. Testosterone supplementation increased the ATP levels, mitochondrial complex V activity and ATP6, ATP8 and ATP5C1 levels in the SN of aged male rats. Furthermore, testosterone deficiency induced by orchiectomy reduced mitochondrial complex V activity, downregulated ATP6 and ATP8 expression and upregulated ATP5C1, ATP5I and ATP5L expression in the SN of adult male rats, while TP replacement reversed these effects. The above results indicated that testosterone enhanced mitochondrial complex V function in the SN of aged male rats by upregulating subunits ATP6 and ATP8. The cylinder test and tapered beam walking test are two methods of detecting coordinated motor behavioral deficits in experimental animals [29, 30]. Performance of these tests depends on the functional status of dopaminergic neurons in the SN [30]. We found that TP supplementation ameliorated the coordinated motor behavioral deficits of aged male rats, suggesting that testosterone enhanced the function of the SN (the brain region rich in dopaminergic neurons). Indeed, TP treatment has been reported to improve dopaminergic activity in aged male rats [31], and testosterone has been demonstrated to support dopaminergic function in adult male rats [32].

The SN is sensitive to energy deficiency, and its normal function depends on a sufficient ATP supply. As energy-generating organelles, mitochondria are crucial for neuronal survival [20, 33]. Mitochondrial function decreases upon aging, as evidenced by the reduced activity of the mitochondrial respiratory chain [34, 35]. Reduced mitochondrial function could be due to deficits in mitochondrial complex V, in addition to complexes I, III and IV [36]. Testosterone is known to induce mitochondrial complexes I, III and IV [13, 14]. However, since ATP synthesis is performed by mitochondrial complex V in the mitochondrial inner membrane [37], we explored whether this complex contributed to the testosterone-induced amelioration of motor behavioral deficits in aged rats.

Mitochondrial complex V is a genetic mosaic consisting of two mtDNA-encoded subunits (ATP6 and ATP8) and 15 nDNA-encoded subunits [25, 26]. Previous studies have indicated that mtDNA is more sensitive to oxidative damage than nDNA [38]. Mutations in mtDNA promote neuronal aging and neurodegenerative disease [39], and the frequency of mtDNA point mutations increases significantly during the course of aging [40]. Thus, in the present study, we screened the mtDNA of *ATP6* and *ATP8* for SNPs. Mutations in these mtDNA-encoded subunit genes can result in a variety of pathologic phenotypes. Dozens of different point mutations in the *ATP6* gene have been found to cause devastating neuromuscular disorders [41]. Mutations in the *ATP8* gene were reported to induce mitochondrial reactive oxygen species generation and secretory dysfunction in conplastic mouse strains [42]. In the present study, 100% DNA sequence identity for both *ATP6* and *ATP8* in the SN was observed among 6Mon, 24Mon and 24Mon-TP rats. Thus, the deficits in mitochondrial complex V in aged male rats may rather have been due to altered transcription or translation of its subunits.

Mitochondrial energy deficiency and reduced mitochondrial complex enzyme activity are important contributors to aging and neurodegenerative disease pathogenesis [43]. Testosterone treatment was reported to enhance mitochondrial energy production in SH-SY5Y cells [44, 45]. Similarly, we found that TP administration to aged rats increased ATP levels in the SN, which may have been associated with the enhanced activity of mitochondrial complex V and the elevated expression of subunits ATP6, ATP8 and ATP5C1 in these rats. To determine which subunits were directly influenced by TP supplementation in aged male rats, we excluded aging-related factors by examining mitochondrial complex V activity and subunit expression in castrated adult male rats. Castration reduced mitochondrial complex V activity in the SN of adult male rats, while TP supplementation reversed this effect. Consistently, a previous study indicated that ATP synthase activity was reduced in castrated adult male rats, but increased four-fold following TP treatment [46]. Therefore, mitochondrial complex V activity may be androgen-dependent.

The present study revealed that 5 of the 17 mitochondrial complex V subunits were influenced by altered testosterone levels in adult male rats. ATP6 and ATP8 mRNA and protein levels were reduced and ATP5C1, ATP5I and ATP5L mRNA and protein levels were increased in castrated adult male rats. Testosterone supplementation of castrated adult male rats restored these parameters to normal levels. In previous studies, altered testosterone levels have had similar effects on mtDNA-encoded subunits in the hippocampus and the SN [13, 14]. Castration-induced testosterone deficiency in adult rats significantly reduced the levels of mtDNA-encoded cytochrome b (a component of mitochondrial complex III) and cytochrome c oxidase subunits 1 and 3 (of mitochondrial complex IV) in the hippocampus [13], and suppressed the expression of NADPH dehydrogenase subunits 1 and 4 (of mitochondrial complex I) in the hippocampus and SN [13, 14].

In combination with the data from TP-treated aged male rats, our data on castrated adult male rats indicated that testosterone supplementation enhanced mitochondrial complex V function in the SN of aged male rats by upregulating subunits ATP6 and ATP8. Previous studies have demonstrated that intracellular androgen receptor-bearing neurons are present in the SN [47, 48], suggesting that specific subsets of neurons in the SN are direct targets of testosterone. A recent study indicated that androgen receptors may be present in mitochondria [49], and several putative androgen receptor binding sequences have been detected in mtDNA, which could be mitochondrial androgen response elements that function as enhancers of mtDNA-encoded genes [50]. Thus, testosterone may upregulate *ATP6* and *ATP8* in the SN via corresponding mitochondrial androgen response elements. Further studies should be performed *in vitro* to explore the potential mechanisms.

Unlike the aged male rats, the castrated adult male rats did not exhibit reduced ATP levels in the SN. We detected ATP levels in tissue blocks, not in mitochondria isolated from tissue blocks. The adult male rat tissues may have had greater compensatory ATP production abilities than the aged male rat tissues due to factors such as 5'-adenosine monophosphate-activated protein kinase (AMPK). AMPK activity is activated on various stress conditions [51] and may be important for maintaining energy balance in eukaryotic cells [52]. AMPK rapidly upregulates metabolic enzymes through direct phosphorylation, thus aligning gene expression with energy requirements at the transcriptional level [53, 54]. When energy is insufficient, AMPK enhances the expression of genes involved in glucose transport, glycolysis [55, 56] and mitochondrial respiration [57]. AMPK also regulates mitochondrial activity and glucose metabolism by phosphorylating peroxisome proliferator-activated receptor gamma coactivator 1-alpha [53, 58]. Reduced AMPK activity has been observed in aged animals [59]; thus, diminished AMPK activity may have contributed to the low ATP levels in the SN in the aged male rats.

In the present study, a paradox was observed in the regulation of ATP5C1 expression in the SN: while TP treatment upregulated ATP5C1 in the SN in aged male rats, it downregulated ATP5C1 in the SN in castrated adult male rats. This discrepancy may have been due to the duration of TP supplementation and the ages of the animals used in this study. TP was administered to the aged male rats for 12 weeks, but was only given to the castrated adult male rats for 4 weeks. Moreover, the aged male rats experienced the natural aging process, while the orchiectomized adult rats received pathological insults. Gonadectomy of adult male rats is known to induce oxidative damage in neurons [14, 60]. The oxidative stress status of animals is a critical determinant of whether androgens will be neuroprotective or neurotoxic to cells [61, 62]. Thus, the response of the SN to TP under different levels of oxidative stress may explain the distinct regulation of ATP5C1 in aged male rats and castrated adult male rats. The TP dosage may also have contributed to the differences in ATP5C1 expression between aged male rats and orchiectomized adult rats. Based on a previous study, we only used a single dosage (1 mg/kg) of TP in the present study [14]; however, the dosage of TP is an important determinant of its neurological effects [14, 63, 64]. Thus, the dose of testosterone should be considered in future analyses of its effects on ATP6, ATP8 and ATP5C1 expression.

A previous study indicated that gonadectomy markedly reduced skeletal muscle ATP levels in both male and female adult rats. Testosterone supplementation restored skeletal muscle ATP levels to normal levels in castrated adult male rats, and also significantly increased the skeletal muscle ATP contents of castrated female rats [65]. Thus, TP supplementation might have the same effects on aged female rats as it had on aged male rats in the present study. Considering that testosterone is an essential hormone for women and that exogenous testosterone enhances cognitive performance and musculoskeletal health in postmenopausal women [66], the effects of TP supplementation on mitochondrial complex V function in female animals should be examined in a future study.

In summary, testosterone supplementation overcame the deficits in mitochondrial complex V in the SN in aged male rats. During aging, testosterone supplementation increased ATP levels and enhanced mitochondrial complex V activity in the SN by upregulating ATP6 and ATP8. Thus, mitochondrial ATP6 and ATP8, as potential testosterone targets, may maintain nigrostriatal dopaminergic function in aged males to some extent.

## MATERIALS AND METHODS

### Animals

Male Sprague-Dawley rats supplied by the Experimental Animal Center of Hebei Medical University were housed at a controlled temperature (22 ± 2°C) on a 12-h light-dark cycle (lights on at 6:00 AM). Food and water were available *ad libitum*. The experimental procedures were approved by the Committee of Ethics on Animal Experiments at Hebei Medical University.

### Experiment 1

Forty-five rats were used to study the effects of testosterone supplementation on mitochondrial complex V function in aged male rats. The rats were randomly divided into the following three groups: the 6-month-old group (6Mon, n=15), the 24-month-old group (24Mon, n=15) and the 24-month-old with TP supplementation group (24Mon-TP, n=15). For the 24Mon-TP group, the rats were subcutaneously injected with TP (1 mg/kg per day) for 12 weeks beginning at the age of 21 months. The body weights of the rats in the 24Mon and 24Mon-TP groups were documented every three weeks. The rats in the 6Mon and 24Mon groups were injected with sesame oil rather than TP. In this experiment, coordinated motor behavior was analyzed, as well as ATP levels and mitochondrial complex V activity in the SN. Then, SNP screening, real-time quantitative polymerase chain reaction (qPCR) and Western blot analyses were performed.

### Experiment 2

Thirty-six adult male rats were used to investigate the effects of testosterone deficiency and testosterone replacement on mitochondrial complex V function. The rats were randomly divided into the following three groups: the sham-operated group (n=12), the gonadectomized group (GDX, n=12) and the GDX with TP administration group (GDX-TP, n=12). The gonadectomy and the sham operation were performed as described previously [3]. For the GDX-TP group, the castrated rats were subcutaneously injected with TP for four weeks (1 mg/kg per day) [14]. The rats in the sham and GDX groups were injected with sesame oil rather than TP. In this experiment, ATP levels and mitochondrial complex V activity in the SN were analyzed. Then, qPCR and Western blot analyses were performed to detect alterations in the mitochondrial complex V subunits in GDX or GDX-TP rats.

### Cylinder test

The apparatus for the cylinder test was a transparent plexiglass cylinder with a diameter of 20 cm and a height of 30 cm. The rats were handled for about 10 min per day for two weeks, and were naive to the apparatus. At the time of the test, the rats were individually placed in the cylinder and were recorded with a digital video camera for 5 min [29]. The number of times the rats contacted the wall with both forelimbs during rearing was documented [2].

### Tapered beam walking test

The tapered beam walking test procedure and score calculation method used in this study were described in detail by Strome et al. [30] and Wang et al. [2], respectively. In brief, 2 cm below a 165-cm-long beam, there was a 2.5-cm-wide ledge on each side, which provided a platform on which the rats could step. The beam was narrower at one end than at the other (6.5 cm wide at the wide end, 1.5 cm at the narrow end). The beam was divided into wide, medium and narrow segments for scoring. The day before the test, the rats were allowed to walk on the tapered beam for training. The following day, each rat was tested five times, and the tests were recorded with a digital video camera. Taking a step with one or two toes of the hindlimb on the main surface of the beam with the other four or three toes overhanging the ledge was scored as a half-foot fault, while stepping with the entire foot on the ledge rather than on the main surface of the beam was scored as a full-foot fault. We used the mean value of the scores for the five tapered beam walking tests from the narrow section of the beam for statistical analysis.

### Sample preparation

The rats were sacrificed by decapitation and their brains were removed quickly. The tissue block containing the SN (between 3.00 mm and 4.08 mm rostral to the interaural axis) [67] was dissected with an ophthalmic scalpel on an ice-cold plate under a stereomicroscope. It was immediately processed for assays of ATP levels and mitochondrial complex V activity, or frozen in liquid nitrogen and stored at -80°C until further use.

### Biochemical analysis

ATP levels were detected according to the protocol of the detection kit (Code A095-1-1, Jiancheng Institute of Biotechnology, China). The SN tissue block was homogenized and centrifuged at 1800 x *g* at 4°C for 10 min. ATP levels in the supernatant were measured spectrophotometrically and normalized to the protein concentration (μM/g protein).

For the detection of mitochondrial complex V activity, mitochondria were isolated with a Tissue Mitochondria Isolation Kit (Code C3606, Beyotime Institute of Biotechnology, China). In brief, SN tissue was homogenized in ice-cold buffer (10 mM 4-(2-hydroxyethyl)-1-piperazineethanesulfonic acid, pH 7.5, including 200 mM mannitol, 70 mM sucrose, 1.0 mM ethylene glycol tetraacetic acid and 2.0 mg/mL serum albumin) and centrifuged at 1000 x *g* at 4°C for 10 min. The supernatant was centrifuged again at 3500 x *g* at 4°C for 10 min to collect the mitochondrial pellet. Mitochondrial complex V activity was measured spectrophotometrically based on the specifications of the detection kit (Code A089-5-1, Jiancheng Institute of Biotechnology), and was normalized to the protein concentration (μM/min/g protein).

### SNP screening

DNA was extracted from the SN tissue block according to the instructions of the TIANamp Genomic DNA Kit (DP304, TIANGEN Biotech, Beijing, China). Targeted DNA fragments were obtained by PCR amplification. The 25-μL PCR reaction included 1 μg of genomic DNA, 10 μM of each primer, 10× PCR buffer, deoxynucleotide triphosphates and TaKaRa Taq DNA polymerase. The primers for *ATP6* or *ATP8* were designed as follows: *ATP6* (5′-AATCATCTCCTCAATAGCCACACT-3′ and 5′-TTGTCAGGAGGCCTAATGATAGGA-3′); *ATP8* (5′-TCACAGCTTCATACCCATTGTACT-3′ and 5′-AGGGATACAATTATTAGGGCTCAG-3′). The PCR cycling conditions included 1 cycle of 95°C for 5 min; 35 cycles of 95°C for 30 s, 55°C for 30 s, and 72°C for 40 s; and 1 cycle of 72°C for 7 min. The PCR-amplified fragments were further processed according to the instructions of the BigDye™ Terminator v3.1 Cycle Sequencing Kit. Target DNAs were sequenced on a 3730XL DNA analyzer (Applied Biosystems, USA). DNAMAN software (Lynnon Biosoft, USA) was used to compare the target DNA sequences among the 6Mon, 24Mon and 24Mon-TP groups.

### qPCR analysis

Total RNA (1 μg) from the SN tissue block was reverse-transcribed using random primers to obtain the first-strand cDNA template. Then, qPCR was performed with 1 μL of cDNA (diluted 1:10), 2 μL of each specific primer and 2×All-in-One^TM^ qPCR Mix (GeneCopoeia Inc., USA) in a final volume of 20 μL. PCR was performed as follows: an initial cycle at 95°C for 15 min, followed by 40 cycles at 95°C for 10 s, 60°C for 20 s, and 72°C for 20 s. Then, the melting curves of the PCR products were analyzed to confirm the specificity of amplification. Gene expression was analyzed using glyceraldehyde-3-phosphate dehydrogenase (*GAPDH*) as an internal control. For all samples, qPCR was performed in triplicate. The relative quantification was performed using the 2^-ΔΔCt^ method. The sets of primers were as follows: *ATP6* (5′-TACCACTCAGCTATCTATAAACCTAAGCA-3′ and 5′-AGTTTGTGTCGGAAGCCTAGAATT-3′), *ATP8* (5′-CCAAACCTTTCCTGCACCTC-3′ and 5′-TGGGGGTAATGAAAGAGGCAAA-3′), *ATP5A1* (5′-AGTGCATGGACTGAGGAACG-3′ and 5′-CCCACTCGTCTGCGAATCTT-3′), *ATP5B* (5′-ACCACCAAGAAGGGCTCGAT-3′ and 5′-CCCACTCGTCTGCGAATCTT-3′), *ATP5C1* (5′-CCAGGAGACTGAAGTCCATCA-3′ and 5′-GGAGCCTGTCCCATACACTCG-3′), *ATP5D* (5′-CACTGTGAATGCGGACTCCT-3′ and 5′-GGATTTGGATCTCAGCCCGT-3′), *ATP5E* (5′-TACTGGCGACAGGCTGGACT-3′ and 5′-TTTATGCTGGTGCCCGAAGT-3′), *ATP5F1* (5′-CTCGCGAGATATGTGAGCGG-3′ and 5′-GTACCGATGTCCCTGTGACC-3′), *ATP5G1* (5′-GACCACGAAGGCACTGCT-3′ and 5′-CGTCTGGCCACCTGGAGA-3′), *ATP5G2* (5′-CTCTACCCGCTCCCTGAT-3′ and 5′-GCCGGACTGCCAAGCAGC-3′), *ATP5G3* (5′-CTGCTCTGATCCGAGCTG-3′ and 5′-ACCGTAGAGCCCTCTCCA-3′), *ATP5O* (5′-AGGTGTCCCTTGCTGTTCTGA-3′ and 5′-TGCCTAGGCGACCATTTTCA-3′), *ATP5H* (5′-CCATCGATTGGGTATCTTTTGTG-3′ and 5′-TCATTCCAGGACTTCAGAGCGTTTC-3′), *ATP5J* (5′-GTCGAACGACTGAAGCGGT-3′ and 5′-GTACTTGCACTGAGTCCCGA-3′), *ATP5I* (5′-TCAAGTTCGGCCGGTACTC-3′ and 5′-CCGCTGCTATTCTTCTCTCCT-3′), *ATP5J2* (5′-GGAACTCAAACACGAACGGC-3′ and 5′-AGGTTAAGCAGATCGGAGCG-3′), *ATP5L* (5′-TACTCGAAGCCTCGATTGGC-3′ and 5′-ACCAGTTTGGGCACTGTGAA-3′) and *GAPDH* (5′-GACTCTTACCCACGGCAAGTT-3′ and 5′-GGTGATGGGTTTCCCGTTGA-3′).

### Western blot analysis

The SN tissue blocks were homogenized in radioimmunoprecipitation assay buffer containing 1% Triton X-100, 0.1% sodium dodecyl sulfate (SDS), 0.5% sodium deoxycholate and protease inhibitors (100 μg/mL phenylmethanesulfonyl fluoride, 30 μg/mL aprotinin and 1 mM sodium orthovanadate), and then sonicated four times for 10 s each. The samples were centrifuged at 12,000 x *g* for 20 min at 4°C, and the supernatants were collected and centrifuged again as before. About 50 μg of protein from the final supernatant was diluted with 4× sample buffer (50 mM Tris, pH 6.8, 2% SDS, 10% glycerol, 0.1% bromophenol blue and 5% β-mercaptoethanol) and heated for 10 min at 95°C. The proteins were separated via SDS polyacrylamide gel electrophoresis on a 12% gel, and were subsequently transferred to a polyvinylidene difluoride membrane (Millipore). The membrane was incubated for 1 h with 5% dry skim milk in Tris-buffered saline containing 0.05% Tween 20 (TBST, pH 7.6) at room temperature. The membrane was rinsed three times with TBST and then incubated overnight with a rabbit anti-ATP6 polyclonal antibody (1:500, A8193, ABclonal), rabbit anti-ATP8 polyclonal antibody (1:200, GTX55993, GeneTex), rabbit anti-ATP5C1 polyclonal antibody (1:500, ARG58368, Arigo Biolaboratories), rabbit anti-ATP5I polyclonal antibody (1:200, HPA035010, Atlas) or rabbit anti-ATP5L polyclonal antibody (1:500, ARG57383, Arigo Biolaboratories) at 4°C. After being washed three times, the membrane was incubated for 1 h with an IRDye 800-conjugated goat anti-rabbit secondary antibody (1:10000, Rockland) at room temperature. The relative band density was analyzed on an Odyssey infrared scanner (LI-COR Biosciences, USA). The densitometry values of ATP6, ATP8, ATP5C1, ATP5I and ATP5L were normalized to those of β-actin, the endogenous control. For all samples, Western blots were performed in triplicate.

### Serum testosterone assay

Trunk blood was collected from the rats in Experiment 1 following decapitation. The harvested trunk blood was allowed to coagulate in open microfuge tubes at room temperature for 30 min. Then, the serum was collected by centrifugation. Serum testosterone levels were measured using a testosterone radioimmunoassay kit based on the manufacturer’s protocol (Tianjin Nine Tripods Medical and Bioengineering Co., Ltd., China).

### Statistical analysis

Data are shown as the mean ± standard deviation (S.D.). Grubb's test was applied to remove possible outliers. The Kolmogorov-Smirnov test was used to determine whether the variables were normally distributed, and Levene’s test was applied to test the homogeneity of variance. One-way analysis of variance (ANOVA) was applied to compare the means of normally distributed variables. If the variance was homogeneous (*P*>0.05, Levene’s test) and the results of one-way ANOVA were significant (*P*<0.05, F-statistic), Tukey’s honestly significant difference post hoc test was used for multiple comparisons. If the variance was unequal (*P*<0.05, Levene’s test), Welch’s F test in one-way ANOVA was used (F′-statistic), and when *P* was *<*0.05, post hoc analyses were done using the Games-Howell procedure [68]. Statistical analyses were performed using the Statistical Package for the Social Sciences 21 software (SPSS Inc., Chicago, IL, USA) and Prism 6 (GraphPad Software Inc., La Jolla, CA, USA). *P<*0.05 was considered statistically significant.

## Supplementary Material

Supplementary Materials 1 and 2
