# Supplementary material for: Testosterone enhances mitochondrial complex V function in the substantia nigra of aged male rats
Source: Aging (Albany NY). 2020 May 23;12(11):10398–414. doi: 10.18632/aging.103265 (PMC7346067; doi:10.18632/aging.103265)
Supplement: Supplementary Materials 1 and 2 [file aging-12-103265-s001..pdf]

SUPPLEMENTARY MATERIALS

```
ORIGIN
6Mon.seq          ATGAACGAAAATCTATTTGCCTCTTTCATTACCCCCACAA    40
24Mon.seq         ATGAACGAAAATCTATTTGCCTCTTTCATTACCCCCACAA    40
24Mon-TP.seq      ATGAACGAAAATCTATTTGCCTCTTTCATTACCCCCACAA    40
Consensus         atgaacgaaaatctatttgcctctttcattacccccacaa

6Mon.seq          TAATAGGTCTACCAATTGTTGTAACCATTATTATGTTCCC    80
24Mon.seq         TAATAGGTCTACCAATTGTTGTAACCATTATTATGTTCCC    80
24Mon-TP.seq      TAATAGGTCTACCAATTGTTGTAACCATTATTATGTTCCC    80
Consensus         taataggtctaccaattgttgtaaccattattatgttccc

6Mon.seq          ATCAATTCTATTCCCATCATCAAAACGCCTAATCAGCAAC   120
24Mon.seq         ATCAATTCTATTCCCATCATCAAAACGCCTAATCAGCAAC   120
24Mon-TP.seq      ATCAATTCTATTCCCATCATCAAAACGCCTAATCAGCAAC   120
Consensus         atcaattctattcccatcatcaaaacgcctaatcagcaac

6Mon.seq          CGACTACACTCATTTCAACACTGACTAATCAAACTTATCA   160
24Mon.seq         CGACTACACTCATTTCAACACTGACTAATCAAACTTATCA   160
24Mon-TP.seq      CGACTACACTCATTTCAACACTGACTAATCAAACTTATCA   160
Consensus         cgactacactcatttcaacactgactaatcaaacttatca

6Mon.seq          TCAAACAAATAATGTTAATCCACACACCAAAAGGACGAAC   200
24Mon.seq         TCAAACAAATAATGTTAATCCACACACCAAAAGGACGAAC   200
24Mon-TP.seq      TCAAACAAATAATGTTAATCCACACACCAAAAGGACGAAC   200
Consensus         tcaaacaaataatgttaatccacacaccaaaaggacgaac

6Mon.seq          CTGAGCCCTAATAATTGTATCCCTAATTATATTTATTGGC   240
24Mon.seq         CTGAGCCCTAATAATTGTATCCCTAATTATATTTATTGGC   240
24Mon-TP.seq      CTGAGCCCTAATAATTGTATCCCTAATTATATTTATTGGC   240
Consensus         ctgagccctaataattgtatccctaattatatttattggc

6Mon.seq          TCAACCAACCTTCTAGGGCTTCTTCCCCATACATTTACCC   280
24Mon.seq         TCAACCAACCTTCTAGGGCTTCTTCCCCATACATTTACCC   280
24Mon-TP.seq      TCAACCAACCTTCTAGGGCTTCTTCCCCATACATTTACCC   280
Consensus         tcaaccaaccttctagggcttcttccccatacatttaccc

6Mon.seq          CTACCACTCAGCTATCTATAAACCTAAGCATAGCCATCCC   320
24Mon.seq         CTACCACTCAGCTATCTATAAACCTAAGCATAGCCATCCC   320
24Mon-TP.seq      CTACCACTCAGCTATCTATAAACCTAAGCATAGCCATCCC   320
Consensus         ctaccactcagctatctataaacctaagcatagccatccc

6Mon.seq          CCTATGAGCAGGAGCCGTAATTCTAGGCTTCCGACACAAA   360
24Mon.seq         CCTATGAGCAGGAGCCGTAATTCTAGGCTTCCGACACAAA   360
24Mon-TP.seq      CCTATGAGCAGGAGCCGTAATTCTAGGCTTCCGACACAAA   360
Consensus         cctatgagcaggagccgtaattctaggcttccgacacaaa

6Mon.seq          CTAAAAAATTCTTTAGCCCACTTCTTACCGCAAGGAACCC   400
24Mon.seq         CTAAAAAATTCTTTAGCCCACTTCTTACCGCAAGGAACCC   400
24Mon-TP.seq      CTAAAAAATTCTTTAGCCCACTTCTTACCGCAAGGAACCC   400
Consensus         ctaaaaaattctttagcccacttcttaccgcaaggaaccc

6Mon.seq          CCATCTCACTAATTCCCATACTAATCATCATCGAAACTAT   440
24Mon.seq         CCATCTCACTAATTCCCATACTAATCATCATCGAAACTAT   440
24Mon-TP.seq      CCATCTCACTAATTCCCATACTAATCATCATCGAAACTAT   440
Consensus         ccatctcactaattcccatactaatcatcatcgaaactat

6Mon.seq          CAGCCTATTTATTCAACCGATAGCACTAGCAGTACGACTA   480
24Mon.seq         CAGCCTATTTATTCAACCGATAGCACTAGCAGTACGACTA   480
24Mon-TP.seq      CAGCCTATTTATTCAACCGATAGCACTAGCAGTACGACTA   480
Consensus         cagcctatttattcaaccgatagcactagcagtacgacta

6Mon.seq          ACAGCAAACATTACAGCAGGCCATCTATTAATGCATCTAA   520
24Mon.seq         ACAGCAAACATTACAGCAGGCCATCTATTAATGCATCTAA   520
24Mon-TP.seq      ACAGCAAACATTACAGCAGGCCATCTATTAATGCATCTAA   520
Consensus         acagcaaacattacagcaggccatctattaatgcatctaa

6Mon.seq          TCGGAGGAGCTACTCTAGTACTTATAGACATCAGCCCACC   560
24Mon.seq         TCGGAGGAGCTACTCTAGTACTTATAGACATCAGCCCACC   560
24Mon-TP.seq      TCGGAGGAGCTACTCTAGTACTTATAGACATCAGCCCACC   560
Consensus         tcggaggagctactctagtacttatagacatcagcccacc

6Mon.seq          AACCGCTACAATTACATTTATTATTCTACTTCTACTTACA   600
24Mon.seq         AACCGCTACAATTACATTTATTATTCTACTTCTACTTACA   600
24Mon-TP.seq      AACCGCTACAATTACATTTATTATTCTACTTCTACTTACA   600
Consensus         aaccgctacaattacatttattattctacttctacttaca

6Mon.seq          GTACTTGAATTTGCCGTAGCCTTAATTCAAGCCTATGTAT   640
24Mon.seq         GTACTTGAATTTGCCGTAGCCTTAATTCAAGCCTATGTAT   640
24Mon-TP.seq      GTACTTGAATTTGCCGTAGCCTTAATTCAAGCCTATGTAT   640
Consensus         gtacttgaatttgccgtagccttaattcaagcctatgtat

6Mon.seq          TCACCCTTCTAGTAAGCCTGTACCTACATGATAACACATA   680
24Mon.seq         TCACCCTTCTAGTAAGCCTGTACCTACATGATAACACATA   680
24Mon-TP.seq      TCACCCTTCTAGTAAGCCTGTACCTACATGATAACACATA   680
Consensus         tcacccttctagtaagcctgtacctacatgataacacata

6Mon.seq          A                                          681
24Mon.seq         A                                          681
24Mon-TP.seq      A                                          681
Consensus         a
```

Supplementary Material 1. Comparison of ATP6 DNA sequences in the substantia nigraamong 6Mon, 24Mon and 24Mon-TP rats. Identity=100%. (n=3 rats/group).

```
ORIGIN
6Mon.seq                ATGCCACAACTAGACACATCCACATGATTTATTACAATCA     40
24Mon.seq               ATGCCACAACTAGACACATCCACATGATTTATTACAATCA     40
24Mon-TP.seq            ATGCCACAACTAGACACATCCACATGATTTATTACAATCA     40
Consensus               atgccacaactagacacatccacatgatttattacaatca

6Mon.seq                TCTCCTCAATAGCCACACTATTTATTTTATTTCAATTAAA     80
24Mon.seq               TCTCCTCAATAGCCACACTATTTATTTTATTTCAATTAAA     80
24Mon-TP.seq            TCTCCTCAATAGCCACACTATTTATTTTATTTCAATTAAA     80
Consensus               tctcctcaatagccacactatttattttatttcaattaaa

6Mon.seq                AATTTCTTCCCAAACCTTTCCTGCACCTCCCTCACCCAAA    120
24Mon.seq               AATTTCTTCCCAAACCTTTCCTGCACCTCCCTCACCCAAA    120
24Mon-TP.seq            AATTTCTTCCCAAACCTTTCCTGCACCTCCCTCACCCAAA    120
Consensus               aatttcttcccaaacctttcctgcacctccctcacccaaa

6Mon.seq                ACTATAGCTACAGAAAAAACGAATAACCCTTGAGAATCAA    160
24Mon.seq               ACTATAGCTACAGAAAAAACGAATAACCCTTGAGAATCAA    160
24Mon-TP.seq            ACTATAGCTACAGAAAAAACGAATAACCCTTGAGAATCAA    160
Consensus               actatagctacagaaaaaacgaataacccttgagaatcaa

6Mon.seq                AATGAACGAAAATCTATTTGCCTCTTTCATTACCCCCACA    200
24Mon.seq               AATGAACGAAAATCTATTTGCCTCTTTCATTACCCCCACA    200
24Mon-TP.seq            AATGAACGAAAATCTATTTGCCTCTTTCATTACCCCCACA    200
Consensus               aatgaacgaaaatctatttgcctctttcattacccccaca

6Mon.seq                ATAA                                        204
24Mon.seq               ATAA                                        204
24Mon-TP.seq            ATAA                                        204
Consensus               ataa
```

Supplementary Material 2. Comparison of ATP8 DNA sequences in the substantia nigra among 6Mon, 24Mon and 24Mon-TP rats. Identity=100%. (n=3 rats/group).
